# RPL35A Downregulation Suppresses Hepatocellular Carcinoma Cell Proliferation via NCAPG2 Inactivation

**DOI:** 10.1002/cam4.70985

**Published:** 2025-06-24

**Authors:** Liang Chen, Yujie Lin, Yu Lai, Yanshan Li, Tao Chen, Xingxi Luo, Yibiao Ye

**Affiliations:** ^1^ Department of Intensive Care Unit, Sun Yat‐Sen Memorial Hospital Sun Yat‐Sen University Guangzhou China; ^2^ Department of Traditional Chinese Medicine, Sun Yat‐Sen Memorial Hospital Sun Yat‐Sen University Guangzhou Guangdong China; ^3^ Department of Gastroenterology, Sun Yat‐Sen Memorial Hospital Sun Yat‐Sen University Guangzhou China; ^4^ Department of Blood Transfusion, Sun Yat‐Sen Memorial Hospital Sun Yat‐Sen University Guangzhou China; ^5^ Department of Hepato‐Billiary Surgery, Sun Yat‐Sen Memorial Hospital Sun Yat‐Sen University Guangzhou China; ^6^ Department of Gastrointestinal Surgery, Sun Yat‐Sen Memorial Hospital Sun Yat‐Sen University Guangzhou China

**Keywords:** cell cycle arrest, HCC, molecular interplay, therapeutic target, tumor modulation

## Abstract

**Background:**

Hepatocellular carcinoma (HCC) is a highly aggressive cancer with a poor prognosis. The molecular mechanisms underlying HCC progression remain poorly understood, prompting the need for novel therapeutic targets. RPL35A, a component of the 60S large ribosomal subunit, is a ribosomal protein involved in ribosome biogenesis and protein synthesis. Beyond its canonical role, increasing evidence suggests that ribosomal proteins such as RPL35A may also exert extraribosomal functions that contribute to tumorigenesis.

**Methods:**

We investigated RPL35A expression in HCC using tissue samples and cell lines. RPL35A levels were correlated with clinicopathological features and prognosis in HCC patients. In vitro, we manipulated RPL35A expression in HCC cells using shRNA lentiviral vectors and assessed its effects on cell proliferation, migration and apoptosis. In vivo, we evaluated tumor growth using xenograft models. Gene expression analysis was conducted to identify downstream targets of RPL35A.

**Results:**

RPL35A was significantly overexpressed in HCC tissues compared to normal liver, correlating with advanced disease stages and poorer prognosis. Knockdown of RPL35A in HCC cells inhibited cell proliferation, migration and invasion, while promoting apoptosis. In vivo, RPL35A silencing reduced tumor growth and size. Gene expression analysis identified NCAPG2 as a key downstream target of RPL35A. NCAPG2 expression was upregulated in HCC, and its knockdown reversed the oncogenic effects of RPL35A. Moreover, RPL35A overexpression increased NCAPG2 levels, promoting tumor progression. These findings suggest that the RPL35A/NCAPG2 axis is crucial in HCC development.

**Conclusions:**

High expression of RPL35A is linked to poor prognosis in hepatocellular carcinoma. The regulation of NCAPG2 by RPL35A may represent a critical mechanism underlying RPL35A‐driven tumor progression. Targeting the RPL35A/NCAPG2 pathway may offer a promising therapeutic strategy for HCC treatment.

**Abbreviations**
DEGs: differentially expressed genes
GEPIA: the Gene Expression Profiling Interactive Analysis
GSEA: Gene Set Enrichment Analysis
HCC: hepatocellular carcinoma
HE: hematoxylin and eosin
MSI: microsatellite instability
NCAPG2: non‐SMC condensin I complex subunit G2
OD: optical density
RPL35A: ribosomal protein L35A
RPs: ribosomal proteins
TMB: tumor mutational burden

## 1 Introduction

Primary liver cancer is a highly aggressive malignancy that includes hepatocellular carcinoma (HCC), constituting a significant proportion, around 75%–85%, of all primary liver cancer cases [1]. Despite advancements in surgical techniques, which have improved overall survival rates to 60%–70% [2, 3], HCC remains a leading cause of cancer‐related deaths worldwide. Alarming statistics from the World Health Organization in 2020 place HCC as the 7th most commonly diagnosed cancer and the 4th leading cause of cancer‐related mortality, which is responsible for over 905,677 new cases and more than 830,180 deaths annually [4]. Given these distressing figures, advanced treatments for HCC, such as sorafenib and Lenvatinib, have made significant progress. However, the increasing resistance of HCC to these molecular targeted therapies, including Lenvatinib, poses a significant challenge to effective treatment [5, 6]. This growing resistance emphasizes the urgent need for new therapeutic approaches to improve treatment outcomes. This study aims to explore the potential role of ribosomal proteins, particularly RPL35A, in HCC. By investigating how RPL35A contributes to tumorigenesis and HCC progression, we hope to provide new insights that could lead to the development of novel therapeutic strategies for liver cancer.

Recently, ribosomal proteins (RPs) have gained significant attention due to their crucial role in ribosome formation and their partnership with rRNA, which is essential for cell survival and overall organism development [7, 8]. Growing research reveals the multifaceted functions of RPs in the context of tumorigenesis [9]. For instance, by interacting with the K homology domain of insulin‐like growth factor 2 mRNA‐binding protein 1 (IGF2BP1), which recognizes and directly binds the 3′‐UTR of MKK6 and MAPK14 mRNA in an m6A‐dependent manner, and promotes translation of core p38 MAPK pathway proteins, RPS15 facilitates ESCC proliferation and metastasis [10]. A number of RPs, including RPL32 [11], RPL15 [12], RPL19 [13], RPL23 [14], etc., have been reported to act as tumor promoters in multiple tumors. On the contrary, others have been shown to act as tumor suppressors, either boosting tumor suppressor pathways or counteracting the actions of oncoproteins, such as RPS6 [15], RPL11 [16], RPL22 [17], etc. Particularly, RPL35A, a gene responsible for coordinating the 60S large ribosomal subunit protein and located on chromosome 3q29‐qter [18], was widely identified to participate in Diamond‐Blackfan anemia [19, 20]. The increased expression of RPL35A in HCC tissues was found to associate with poor patient prognosis [13]. Meanwhile, knockdown of RPL35A significantly suppress cell proliferation, migration, enhance apoptosis and arrest cell cycle of gastric cancer, also suggesting a role of RPL35A as a tumor promoter [21]. However, the intricate mechanisms orchestrated by RPL35A in HCC remain largely unknown.

This study provides robust confirmation of RPL35A's upregulation in HCC tissues relative to adjacent non‐cancerous tissues. Furthermore, it establishes a strong correlation between heightened RPL35A expression and unfavorable patient outcomes, a finding consistently observed in both TCGA datasets and our own HCC samples. Notably, the downregulation of RPL35A yielded significant consequences, including the inhibition of HCC proliferation, an increased apoptosis rate, diminished cell migration and invasion and a pronounced impediment to tumorigenesis. Additionally, our investigation identified NCAPG2 as a downstream molecule regulated by RPL35A, and the attenuation of NCAPG2 expression effectively restored cellular functions that were initially influenced by RPL35A overexpression. In conclusion, our findings shed light on the potential therapeutic significance of RPL35A in the therapeutic landscape of HCC.

## 2 Methods

### 2.1 Patient Tissue Collection and Immunohistochemistry

HCC and corresponding normal tissues, comprising 89 samples, were obtained from Shanghai Outdo Biotech CO.Ltd and assembled into a tissue microarray. Detailed patient information was meticulously recorded, and prior informed consent was acquired before surgical procedures. All methods were approved by the Institutional Review Board at SUN YAT‐SEN Memorial Hospital.

For the immunohistochemical (IHC) analysis, formalin‐fixed paraffin‐embedded tissue sections were deparaffinized, rehydrated through graded ethanol and subjected to antigen retrieval using citrate buffer (pH 6.0) at 95°C for 20 min. Endogenous peroxidase activity was blocked using 3% hydrogen peroxide for 10 min. The sections were then incubated with a primary antibody against RPL35A (1:100 dilution, Biorbyt, Cambridge, UK; catalog no. orb513214) overnight at 4°C. After washing, the sections were incubated with a secondary antibody and visualized using DAB substrate, followed by counterstaining with hematoxylin.

### 2.2 Cell Culture and Transfection

HCC cell lines, purchased from BeNa Technology, including BEL‐7404 and SK‐HEP‐1, were incubated in DMEM (Thermo Fisher Scientific, USA) and supplemented with 10% FBS (Thermo Fisher Scientific) at an atmosphere of 5% CO_2_ at 37°C.

For transfection, HCC cells were evenly distributed in 24‐well plates and incubated overnight. Subsequently, they were transfected using lentiviral vectors, which carried either small interfering RNA targeting RPL35A or NCAPG2, an overexpression sequence for RPL35A, or a control lentivirus, all of which were produced by Shanghai Genechem Co. Ltd. The transfection process utilized the HitransG (A&P Set) reagent, also provided by Shanghai Genechem Co. Ltd. Post‐transfection effects were assessed using fluorescence inverted microscopy (Olympus, Japan), as well as RT‐qPCR and Western blot techniques.

### 2.3 RNA Processes and Quantitative PCR

Total RNA from cells was isolated using the TRIzol reagent (Thermo Fisher Scientific). RNA concentration and purity were determined using a Multiskan SkyHigh microplate reader (Thermo Fisher Scientific), based on the optical density (OD) ratio at 260/280 nm. The isolated RNAs were then reverse‐transcribed into cDNAs employing a cDNA Reverse Transcription Kit (Applied Biosystems, USA). qRT‐PCR was carried out on an ABI7500 system (Applied Biosystems) with a protocol of 95°C for 10 min, followed by 36 cycles of 95°C for 10 s and 60°C for 30 s. Gene expression was quantified using the 2−∆∆Ct method, with GAPDH serving as the internal reference. All primers were procured from Sangon Biotech (Shanghai, China), and their sequences are provided as follows,

RPL35A: GAAGGTGTTTACGCCCGAGAT, CGAGTTACTTTTCCCCAGATGAC;

NCAPG2: GAAGAAGGTGACTGGGGAACT, GAAGAAGGTGACTGGGGAACT;

GAPDH: TGACTTCAACAGCGACACCCA, CACCCTGTTGCTGTAGCCAAA.

### 2.4 Western Blot

Cells were rinsed twice with PBS and lysed using RIPA buffer with added components: 0.25% Sodium deoxycholate, 0.1% SDS, 1% Triton X‐100, 1 mM Na3VO4, 10 mM NaF, and a protease inhibitor (Roche, USA). After a 10‐min incubation on ice, lysates were collected by scraping and then centrifuged at 12,000 rpm for 30 min at 4°C. The clarified supernatants were gathered, and protein concentrations were determined using the Bradford assay (Bio‐Rad). For electrophoresis, 20 μg of cell lysate and 10 μL of exosomes were loaded onto a 10% SDS‐PAGE gel. Proteins were then transferred onto a 0.45 μm PVDF membrane (Millipore, USA) at 400 mA for 1 h. The membrane was blocked in 0.1% TBST containing 5% nonfat milk for an hour, followed by overnight incubation at 4°C with primary antibodies (anti‐RPL35A, 1:2000, ab241070, Abcam, USA; anti‐GAPDH, 1:3000, AP0063, Bioworld, China) diluted in 0.1% TBST with 5% BSA. The next day, after incubation with HRP‐conjugated secondary antibodies (Goat‐anti‐rabbit IgG, 1:3000, A0208, Beyotime, China) in 0.1% TBST with 5% nonfat milk at room temperature for an hour, protein bands were visualized using ECL reagent (PerkinElmer, USA).

### 2.5 Colony Formation

Following transfection, the infected cells were plated in 6‐well plates at a density ranging from 400 to 1000 cells per well. After a 2‐week incubation period, the cells were fixed using 4% paraformaldehyde for 30 min at room temperature. Subsequently, they were stained with 500 μL of Giemsa solution (Dingguo Changsheng, Beijing, China) for a duration of 20 min. All colonies were then visualized, captured as images and quantified.

### 2.6 Cell Apoptosis Detection

HCC cells from each group were collected and prepared according to the Annexin V Apoptosis Detection kit instructions (Vazyme Biotech, Nanjing, China). Cells were resuspended in 100 μL of loading buffer. In the experimental group, cells were added with 5 μL Annexin V solution and then incubated in the dark at room temperature for 10 min before being mixed with 400 μL of Loading Buffer. Finally, apoptosis rates were assessed using flow cytometry (Beckman, USA).

### 2.7 Wound Healing Assay

The migration capabilities of HCC cells were assessed using a wound healing assay. In essence, after allowing the transfected BEL‐7404 and SK‐HEP‐1 cells to fully adhere in 6‐well plates, a scratch was introduced using a pipette tip. After clearing away any cellular debris, the cells were incubated for an additional 24 h. Migration was evaluated by comparing the wound width at the initiation (0 h) to that at 24 h post‐scratch.

### 2.8 Transwell Assay

Transwell invasion assays utilized 24‐well Transwell filters with an 8 μm pore size (Costar 3422, China), which were primed with 30 μg of Matrigel (BD Biosciences, USA). BEL‐7404 cells (2 × 10^4^) and SK‐HEP‐1 cells (5 × 10^4^) were placed in the top chamber with serum‐free medium, while the bottom chamber was filled with DMEM supplemented with 10% FBS. After a 48‐h incubation, cells that had not migrated through the filter were gently wiped away using a cotton swab. The cells that had successfully migrated to the bottom chamber were then fixed with 4% paraformaldehyde and stained using 0.5% crystal violet. The total number of migrated cells was determined by counting cells in five random fields under 40× magnification for each filter.

### 2.9 In Vivo Tumorigenesis

This study's animal experiments were greenlit by the Institutional Animal Care and Use Committee at SUN YAT‐SEN Memorial Hospital's Research Center for Drug Safety Evaluation and were meticulously conducted in line with their prescribed guidelines. BALB/c nude male mice, aged 4–5 weeks, were sourced from Shanghai Lingchang Laboratory Animal Technology Co. Ltd. For the study, we injected 1 × 10^7^ SK‐HEP‐1 cells, either transfected with a control or shRPL35A lentivirus, into the right dorsal region of these mice. Every 7 days, we gauged the tumor's size and the mice's weight. We calculated the tumor volume using the formula: length × width^2^/2 (mm^3^). At the study's end on day 30, after anesthetizing the mice with pentobarbital sodium, they were humanely euthanized through cervical dislocation. We then harvested the tumor tissues, which underwent subsequent hematoxylin and eosin (HE) staining and immunohistochemical analysis for ki67 with the antibodies (anti‐ki67, 1:200, Ab16667; goat‐anti‐rabbit IgG H&L (HRP), 1:400, ab6721) purchased from Abcam.

### 2.10 Gene Expression Microarray

Total RNA was reverse‐transcribed using the T7 Oligo(dT) primer and amplified with the Illumina TotalPre RNA Amplification Kit (Ambion, USA) to produce cDNA with a T7 promoter sequence. The synthesis of the second cDNA strand transformed the single‐stranded cDNA into a double‐stranded DNA template, employing DNA polymerase, and RNase H. This dsDNA then underwent in vitro transcription to produce multiple biotinylated cRNA copies using T7 RNA polymerase. The resultant cRNA was hybridized to Illumina Human HT‐12 v4 BeadChips at 58°C for 16 h. Post‐hybridization, the BeadChip was stained with streptavidin‐Cy3 and scanned with HiScan SQ (Illumina). Data were processed using BeadStudio software.

The acquired intensity data from the Illumina Human HT‐12 v4 BeadChips was assessed with Partek software for mRNA analysis. Background‐corrected signals underwent quantile normalization. Differentially expressed genes (DEGs) were subjected to functional annotation using the DAVID platform (https://david.ncifcrf.gov) to understand their roles in GO terms and KEGG pathways. Moreover, to discern significant gene sets associated with tumor and normal phenotypes, we used the Gene Set Enrichment Analysis (GSEA) tool. The hallmark gene set from MSigDB 3.0 was selected for the enrichment analysis, and results were validated based on 100 permutations, considering a corrected *p*‐value < 0.05 and an FDR < 0.25 as significant.

### 2.11 TCGA Analysis

The expression levels of RPL35A and NCAPG2 in both normal and tumor patients were discerned through an analysis of the TCGA database using the Gene Expression Profiling Interactive Analysis (GEPIA) tool (available at http://gepia2.cancer‐pku.cn/#index). Survival outcomes from TCGA‐LIHC were mapped out using the Kaplan–Meier Plotter. HCC tumor samples were categorized into two groups based on RPL35A expression, and the differentially expressed genes were screened by the limma method. These genes were then subjected to further analysis, including GO, KEGG, and GSEA enrichment assessments.

### 2.12 Statistical Considerations

Statistical analyses, comprising unpaired *t*‐test, one‐way ANOVA, Pearson and Spearman correlation evaluations, and the chi‐square test, were conducted using GraphPad Prism8 software. Experiments were independently replicated at least three times, with data represented as the mean ± SD. A value of *p* < 0.05 was deemed statistically significant. Significance levels were indicated as follows: *p < 0.05, **p < 0.01, and ***p < 0.001.

## 3 Results

### 3.1 RPL35A Expression and Implications in HCC Prognosis

**FIGURE 1:**
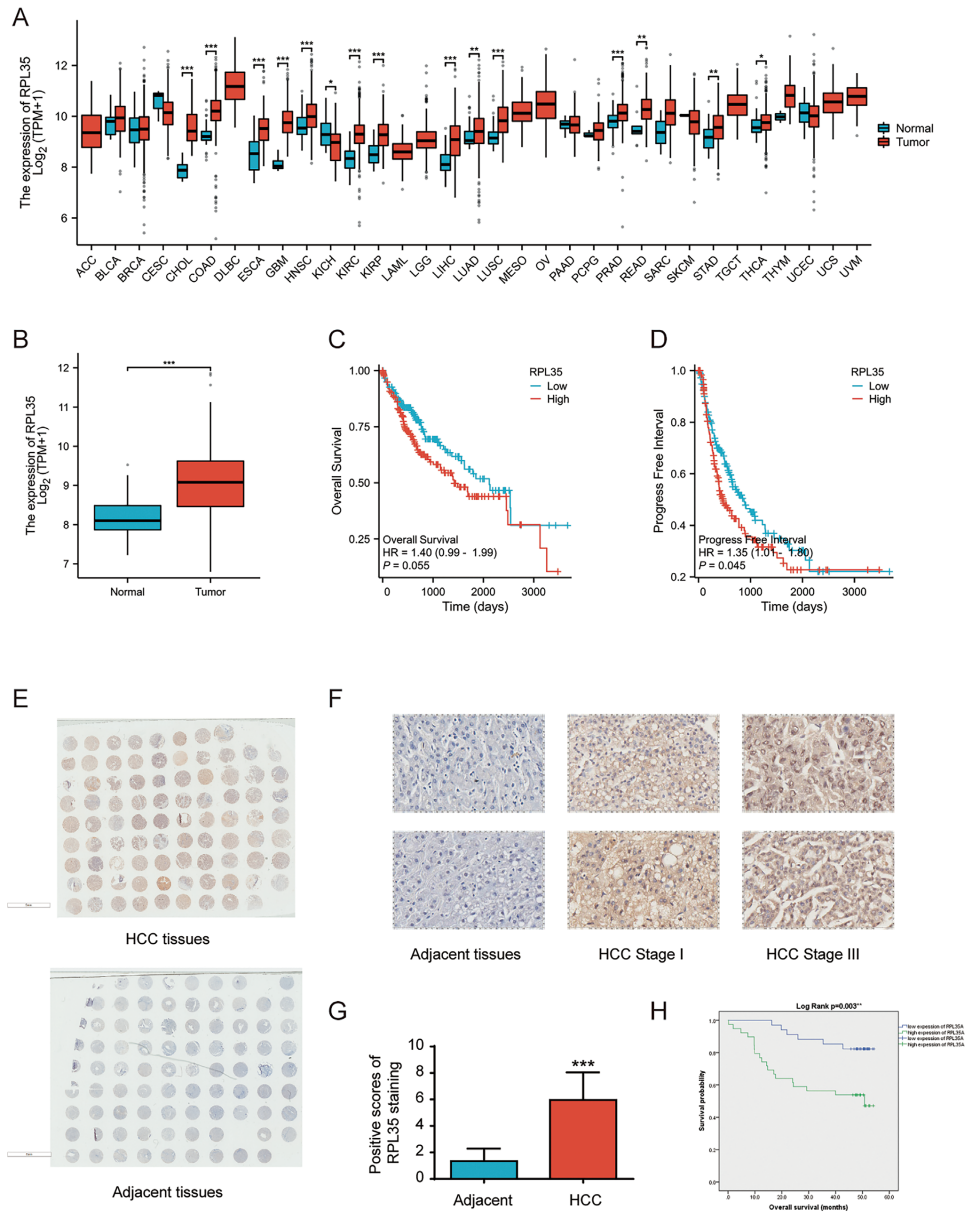
Elevated RPL35A expression associated with hepatocellular carcinoma progression and adverse prognosis. (A) RPL35A mRNA expression demonstrated upregulation in various tumor tissues when compared to normal samples, as per data from The Cancer Genome Atlas (TCGA) database. (B) HCC tumor tissues exhibited increased RPL35A expression compared to adjacent normal tissues based on TCGA database. Data are presented as mean with standard deviation. ***p < 0.001. (C) Kaplan–Meier survival analysis illustrates the correlation between RPL35A expression and overall survival in HCC patients according to TCGA database. (D) Kaplan–Meier survival analysis reveals the relationship between RPL35A expression and progression‐free interval among HCC patients according to TCGA database. (E) Photographs of the tissue microarray following immunohistochemistry for RPL35A are presented. (F) Exemplary images of immunohistochemical staining of RPL35A in HCC and adjacent tissues are presented. (G) The staining score for RPL35A in HCC tissues was significantly higher compared to adjacent tissues. Data are presented as mean with standard deviation. *p < 0.05, **p < 0.01, ***p < 0.001. (H) Kaplan–Meier survival analysis demonstrates the association between RPL35A expression and overall survival in HCC patients in our own dataset.

To investigate the relationship between RPL35A expression and various cancers, we initiated a comprehensive analysis of RPL35A expression across different tumors, revealing notable dysregulation of RPL35A in liver hepatocellular carcinoma, as seen in Figure 1A. Upon comparing the expression levels of RPL35A in 371 HCC samples to 50 normal liver tissues using a *T*‐test, we observed a significant upregulation of RPL35A in HCC samples (Figure 1B). For further insights, we divided HCC patients into high and low RPL35A expression groups based on the median RPL35A counts. Subsequent survival analysis, in correlation with prognosis data provided in TCGA, indicated a borderline difference in overall survival between the two groups (Figure 1C, *p* = 0.055). A more pronounced difference was noted in the Progress‐Free Interval (Figure 1D, *p* = 0.045), suggesting that higher RPL35A levels might be indicative of a poorer prognosis.

To bolster this assertion, we utilized a tissue microarray consisting of both HCC and adjacent normal tissues, and carried out IHC staining. A comprehensive view of the microarray staining is depicted in Figure 1E. Selective images, presented in Figure 1F, unveil a progressive increase in RPL35A protein levels from para‐carcinoma tissues to advanced HCC stages. Statistical analyses consistently revealed a robust association between RPL35A overexpression in HCC and the severity of the disease, as seen in Table 1 and Figure 1G. Reinforcing this, the Kaplan–Meier survival analysis highlighted a negative relationship between RPL35A expression levels and patient survival, further emphasizing its prognostic relevance (Figure 1H). Taken together, the pronounced overexpression of RPL35A in HCC underscores its potential as a target for therapeutic intervention.

**TABLE 1:** Relationship between RPL35A expression and tumor characteristics in patients with hepatocellular carcinoma.

Features	No. of patients	RPL35A expression		*p*
		Low	High	
All patients	84	38	46	
Age (years)				
< 59	41	15	26	0.099
≥ 59	42	23	19	
Gender				
Male	69	30	39	0.352
Female	14	8	6	
Grade				
II	42	24	18	0.026*
III	40	13	27	
Tumor size				
< 6 cm	41	21	20	0.329
≥ 6 cm	42	17	25	
Stage				
I	27	18	9	0.004**
II	39	16	23	
III	16	4	12	
IV	1	0	1	
T Infiltrate				
T1	27	18	9	0.006**
T2	40	16	24	
T3	5	1	4	
T4	11	3	8	

*Note:* ‘T Infiltrate’ refers to the T classification within the TNM staging system, which describes the extent of the primary tumor in hepatocellular carcinoma (HCC). Specifically, the T staging in our study follows the criteria outlined in the 8th edition of the American Joint Committee on Cancer (AJCC) staging system, which is internationally recognized. The T categories are defined as: T1: A solitary tumor without vascular invasion. T2: A solitary tumor with vascular invasion, or multiple tumors none larger than 5 cm. T3: Multiple tumors with at least one tumor larger than 5 cm. T4: Tumor(s) with direct invasion into adjacent organs (excluding the gallbladder) or with perforation of the visceral peritoneum. Clinical staging (Stage I–IV) was determined by combining tumor characteristics (T), regional lymph node involvement (N), and distant metastasis (M), also according to the AJCC 8th edition: Stage I: T1N0M0. Stage II: T2N0M0. Stage III: T3 or T4, or any T with N1, but M0. Stage IV: Any T, any N, with distant metastasis (M1). *p < 0.05, **p < 0.01.

### 3.2 Downregulation of RPL35A Suppressed HCC Proliferation and Metastasis In Vitro

**FIGURE 2:**
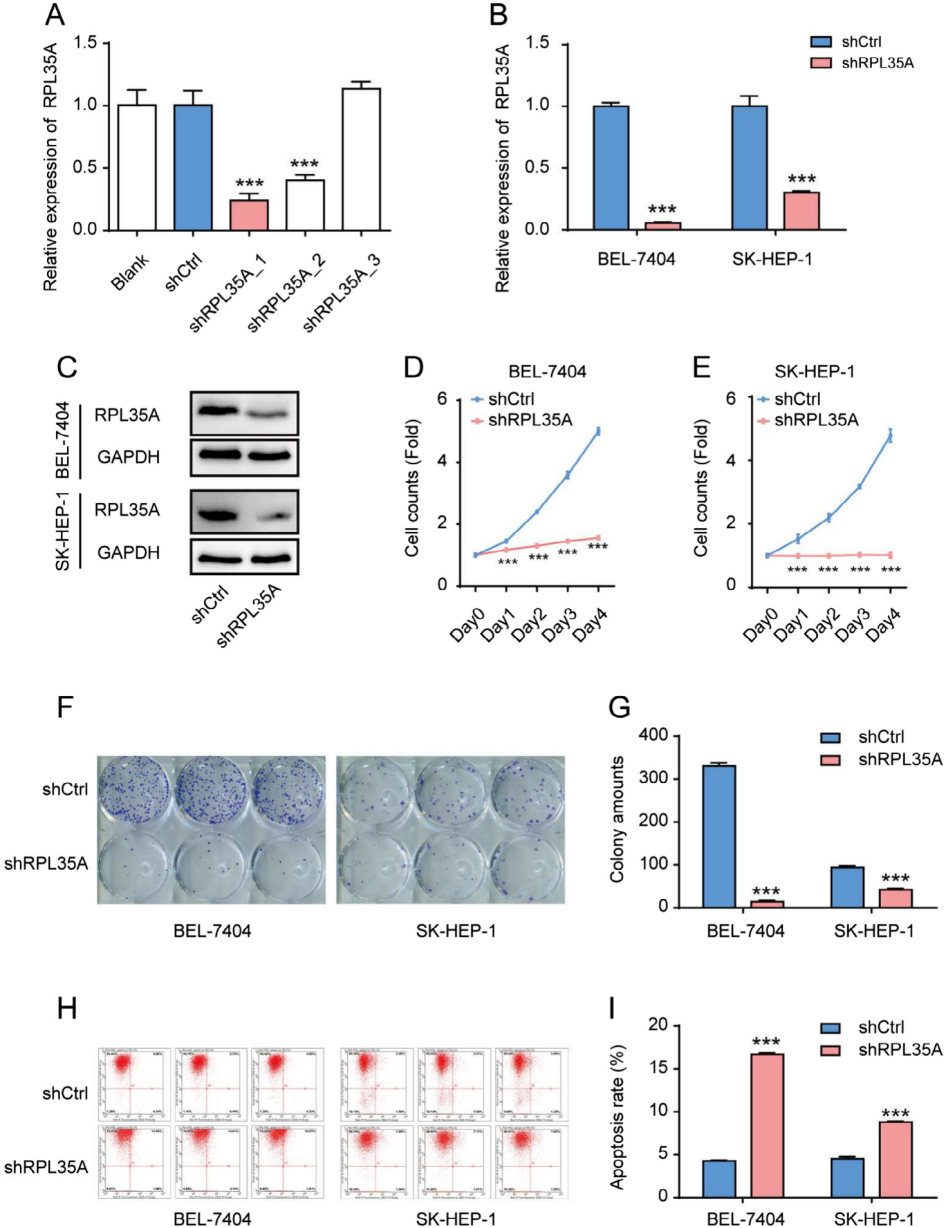
Knockdown of RPL35A inhibits proliferation and enhances apoptosis in HCC cells. (A) Transfection of BEL‐7404 cells with three shRNAs was conducted, and the efficiency of knockdown was assessed using RT‐qPCR. (B) Knockdown of RPL35A with shRPL35A_1 lentivirus was performed, and RPL35A expression was quantified in both BEL‐7404 and SK‐HEP‐1 cells. (C) Downregulation of RPL35A protein in HCC cells was confirmed through western blot analysis. (D, E) Cell counts assessed via the Celigo method confirmed decreased cell viability in shRPL35A‐treated HCC cells compared to the shCtrl group. (F) Colony formation assay further verified reduced cell viability in shRPL35A HCC cells relative to the shCtrl group. (G) The number of colonies corroborated the diminished cell viability in shRPL35A HCC cells compared to the shCtrl group. (H) Annexin V staining and subsequent flow cytometric analysis depicted changes in apoptosis rates following RPL35A knockdown. (I) Statistical analysis of cell apoptosis rates after shRPL35A and shCtrl virus infections, demonstrating a significant increase in apoptosis rates following RPL35A knockdown. Data are represented as the mean with standard deviation. ***p < 0.001.

**FIGURE 3:**
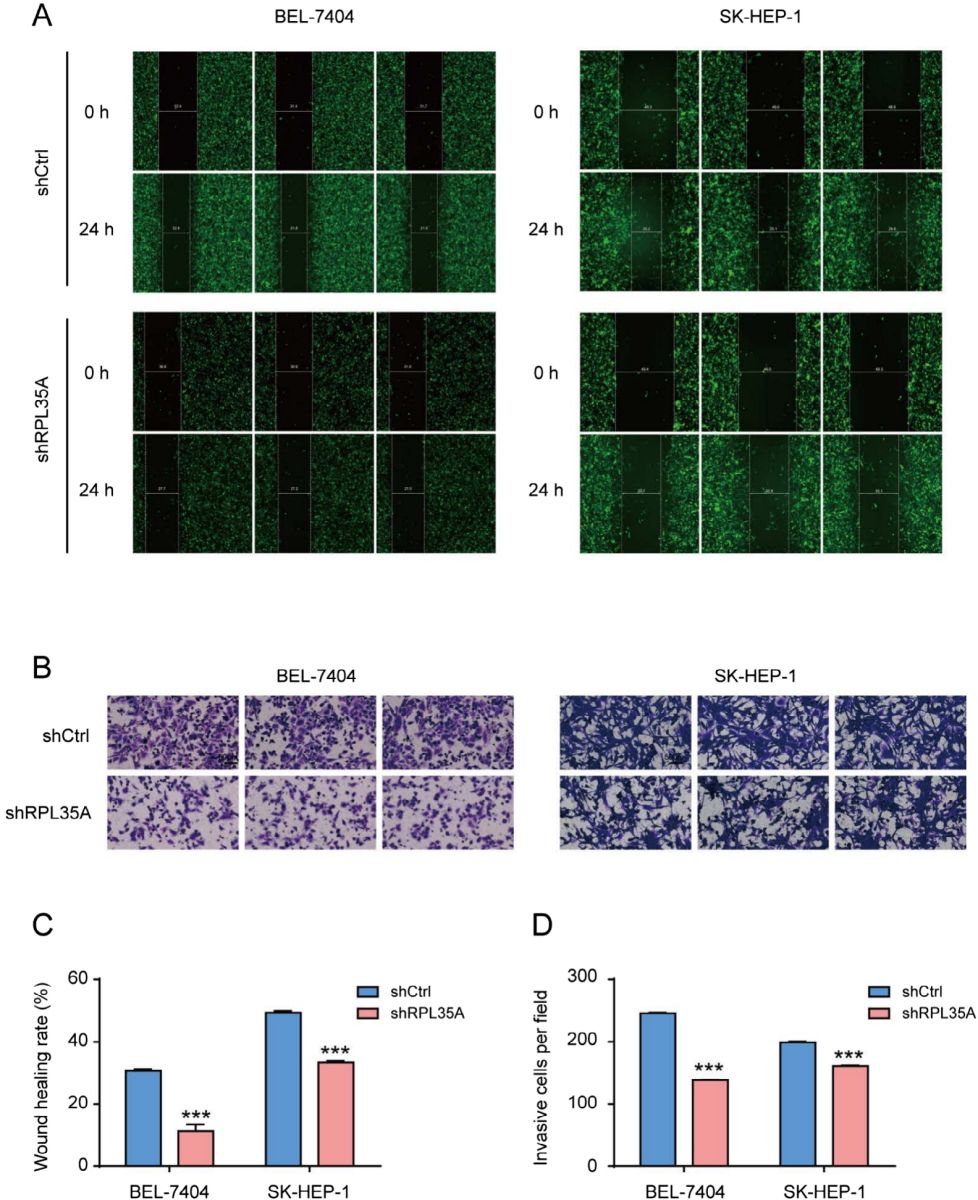
RPL35A knockdown reduces migration and invasion in HCC cells. (A) Images of cells following scratch assays were captured to assess the wound healing capacity of cells with altered RPL35A expression. (B) Statistical analysis of cell wound healing rates post shRPL35A and shCtrl virus infections, revealing a significant decrease in cell migration following RPL35A knockdown. (C) Images of cells that traversed Matrigel and Transwell membranes were stained to evaluate the invasive potential of cells with modified RPL35A expression. (D) Statistical analysis of the number of cells that traversed the chamber following shRPL35A and shCtrl virus infections, demonstrating a significant reduction in cell invasion after RPL35A knockdown. Data are represented as the mean with standard deviation. ***p < 0.001.

To manipulate the expression of RPL35A in HCC cells, we constructed three shRNA lentiviruses. Following transfection, RT‐QPCR analysis showed that the first shRNA most effectively downregulated RPL35A and was therefore selected for subsequent experiments (Figure 2A). The efficiency of RPL35A knockdown in both BEL‐7404 and SK‐HEP‐1 cells was then confirmed using RT‐QPCR and Western blot analyses (Figure 2B,C). Following RPL35A silencing, there was a noticeable decline in HCC cell proliferation, as demonstrated by the Celigo cell counting assay (Figure 2D,E). In tandem, the capacity for colony formation was significantly diminished in the shRPL35A group (Figure 2F,G). Using Annexin V‐APC staining in conjunction with flow cytometry, we observed a marked increase in apoptosis in cells infected with shRPL35A (Figure 2H,I). Moreover, wound healing assays highlighted that the suppression of RPL35A hindered HCC cell migration (Figure 3A,C). Similarly, based on Transwell invasion assays, the downregulation of RPL35A also curtailed HCC invasion (Figure 3B,D). Collectively, these findings underscore the oncogenic role of RPL35A in HCC.

### 3.3 Downregulation of RPL35A Suppressed HCC Tumorigenesis In Vivo

**FIGURE 4:**
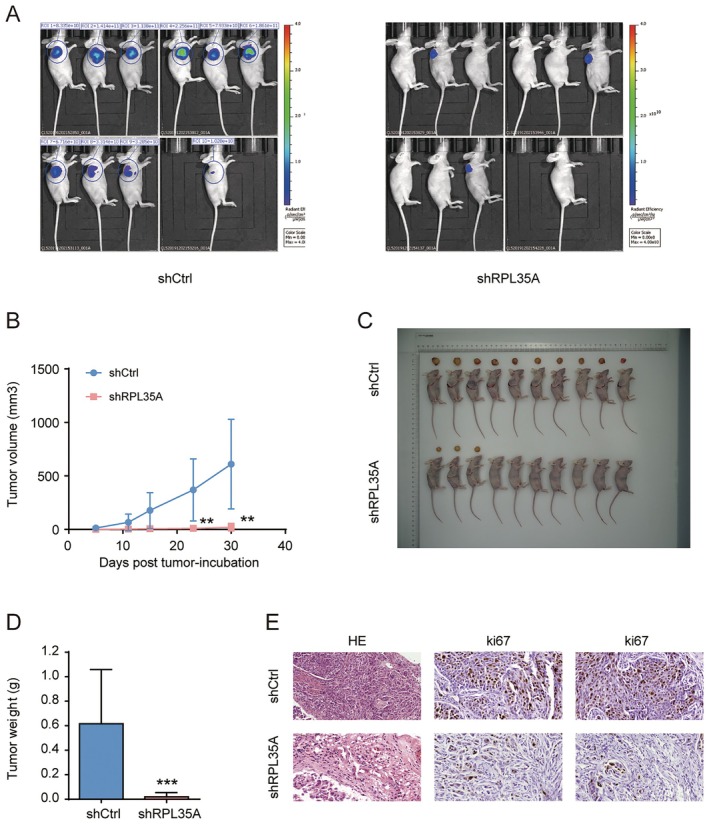
RPL35A knockdown suppresses HCC tumorigenesis in vivo. (A) Live fluorescence images of xenografts taken 28 days post‐injection of SK‐HEP‐1 cells were presented. (B) Measurement of tumor volumes confirmed inhibited tumor growth in mice injected with shRPL35A‐transfected cells. (C) Depictions of sacrificed mice and excised tumor tissues were presented. (D) Tumor weight significantly reduced in the shRPL35A group compared to the shCtrl group. (E) IHC staining of isolated tumor tissues corroborated the downregulation of ki67 in the shRPL35A group compared to the shCtrl group. Data are represented as the mean with standard deviation. **p < 0.01, ***p < 0.001.

To delve deeper into the role of RPL35A in the progression of HCC in vivo, we established a xenograft tumor model by subcutaneously injecting SK‐HEP‐1 cells, with varied RPL35A expressions, into the right flank of nude mice. Four weeks post‐model establishment, we conducted in vivo fluorescence imaging on both control mice (shCtrl) and those injected with shRPL35A‐infected cells. Notably, mice with RPL35A knockdown exhibited lower tumorigenicity and reduced fluorescence intensity (Figure 4A). When comparing tumor volume between the groups, we observed that RPL35A depletion significantly decelerated tumor growth (Figure 4B). On day 31 post‐cell injection, the mice were euthanized, and the excised tumors were photographed alongside them, as depicted in Figure 4C. Further weighing of the tumor tissues revealed a significant reduction in tumor weight following RPL35A knockdown (Figure 4D). Histological examinations, including HE staining and IHC staining of the tumor tissues, indicated decreased Ki67 expression upon RPL35A silencing. In summary, our findings suggest that the knockdown of RPL35A significantly impedes HCC progression in mice.

### 3.4 Decoding RPL35A's Downstream Regulatory Network in HCC

**FIGURE 5:**
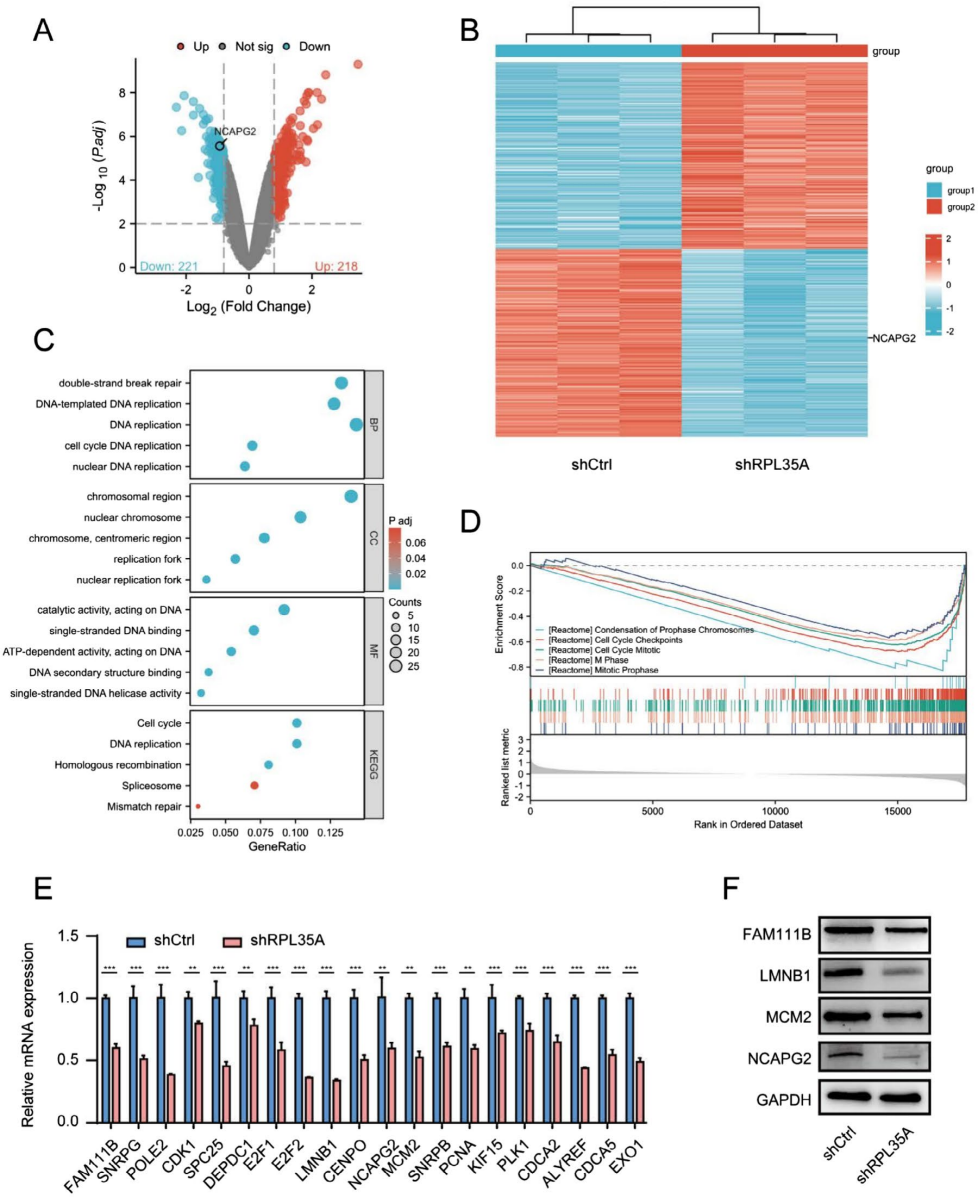
NCAPG2 was downregulated in SK‐HEP‐1 cells after silencing of RPL35A. (A) A mRNA microarray analysis was conducted to identify dysregulated genes after RPL35A knockdown, and the volcano plot illustrates the differentially expressed genes. (B) A heatmap displays differentially expressed genes. (C) Functional enrichment analysis through Gene Ontology (GO) and Kyoto Encyclopedia of Genes and Genomes (KEGG) pathways highlights the pathways that are suppressed after RPL35A knockdown. (D) Gene Set Enrichment Analysis (GSEA) reveals the deactivation of pathways associated with the cell cycle after RPL35A knockdown. (E) Validation of the downregulated genes, as selected by the microarray, was performed using RT‐qPCR. (F) Western blot analysis further validates the downregulation of selected genes, as identified by the microarray. Data are shown as the mean with standard deviation. **p < 0.01, ***p < 0.001.

To further elucidate the regulatory mechanism of RPL35A, we employed a Gene Expression Array on SK‐HEP‐1 cells, either treated with RPL35A shRNA or left untreated. By setting the criteria of a log2Fold Change greater than 0.8 or less than −0.8, combined with an adjusted *p*‐value less than 0.01, we identified 218 genes upregulated and 221 genes downregulated following RPL35A knockdown (Figure 5A). The differentially expressed genes (DEGs) are visualized in a heatmap as shown in Figure 5B. Subjecting these DEGs to GO and KEGG functional enrichment analysis, we observed inactivated pathways, including ‘DNA replication’ and relevant pathways (Figure 5C). Further GSEA analysis revealed that post‐shRPL35A infection, several cell cycle‐related pathways like ‘Mitotic Prophase,’ ‘M phase,’ ‘Cell Cycle Mitotic,’ ‘Cell cycle Checkpoints’ and ‘Prophase Chromosome’ were notably inhibited. This provides some insight into how RPL45A may regulate cell proliferation.

Furthermore, after separating TCGA HCC patients into high and low RPL35A expression groups based on the median expression of RPL35A, we analyzed gene expressions for both categories. Top 20 upregulated genes in high RPL35A group were presented in Figure S1A. GO enrichment analysis was performed on genes highly expressed in the RPL35A high‐expression group, revealing several promoted pathways, including ‘DNA‐binding transcription activator activity,’ as shown in Figure S1B. Additionally, GSEA enrichment analysis indicated that elevated RPL35A expression enhanced pathways related to ‘DNA replication’ and ‘Ribosome’ (Figure S1C).

Based on the chip results, we validated the expression of genes downregulated after shRPL35A infection. We found that both the RNA and protein levels of FAM111B, LMNB1, MCM2 and NCAPG2 notably decreased when RPL35A expression was reduced (Figure 5E,F). Additionally, in the TCGA database, a significant positive correlation was observed between NCAPG2 and RPL35A expression as determined by Spearman analysis (Figure S1D). These findings offer insights and evidence to further understand the oncogenic mechanism of RPL35A.

### 3.5 NCAPG2: Integral to RPL35A‐Driven HCC Progression

**FIGURE 6:**
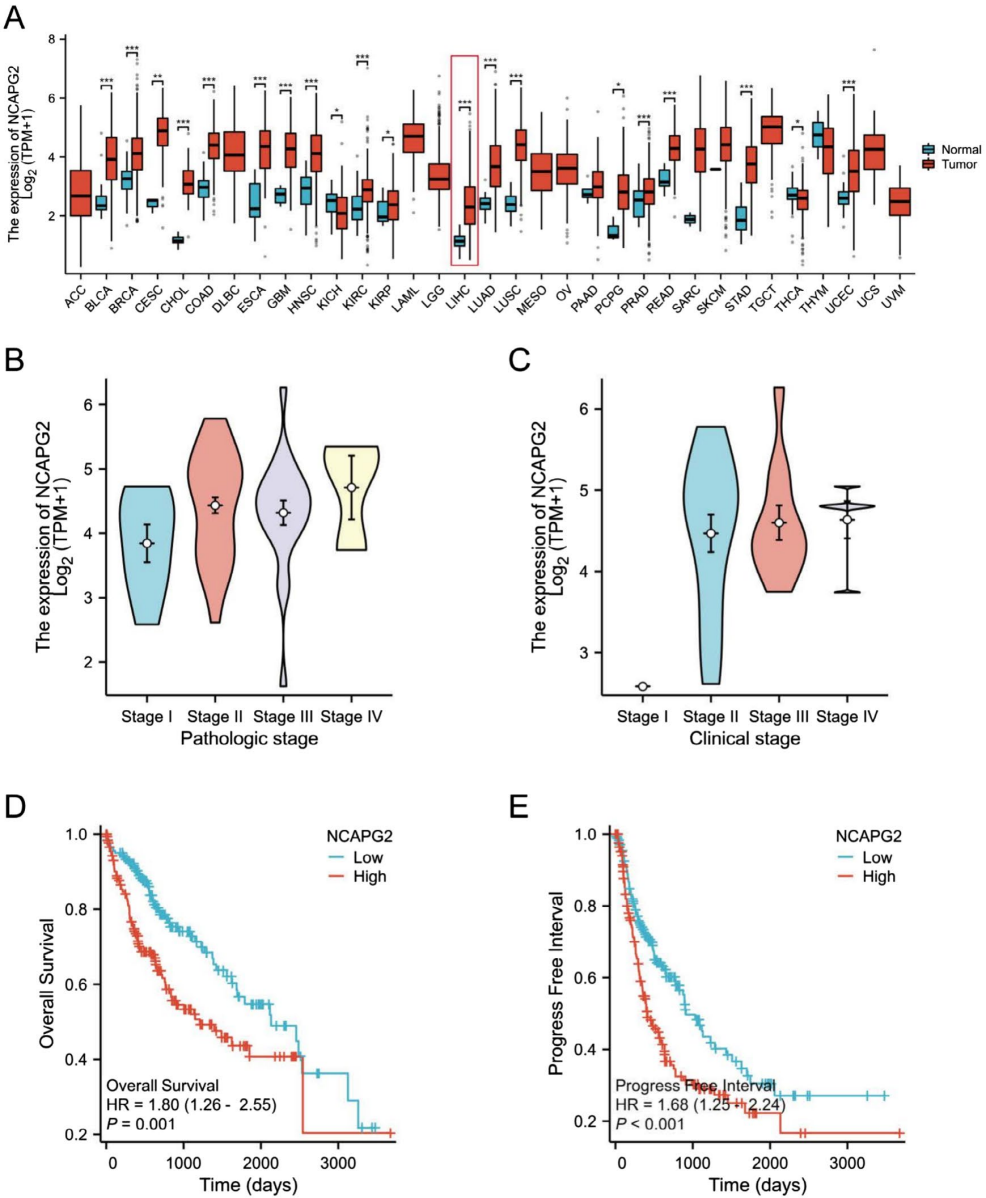
Elevated NCAPG2 expression is associated with hepatocellular carcinoma progression and adverse prognosis. (A) Expression levels of NCAPG2 mRNA were notably elevated in various tumor tissues when compared to their respective normal samples, as indicated by data from the TCGA database. (B) In the context of pathologic staging, expression of NCAPG2 was significantly higher in stages II, III, or IV compared to stage I. (C) Concerning clinical staging, expression of NCAPG2 was substantially higher in stages II, III, or IV compared to stage I. (D) Kaplan–Meier survival analysis underscores the correlation between NCAPG2 expression and overall survival among HCC patients. (E) Kaplan–Meier survival analysis highlights the relationship between NCAPG2 expression and progression‐free interval in HCC patients. Data are shown as the mean with standard deviation. *p < 0.05, **p < 0.01, ***p < 0.001.

To identify potential downstream targets of RPL35A, we first leveraged the TCGA database to examine gene expressions influenced by RPL35A silencing. Notably, NCAPG2 was consistently found to be upregulated in multiple cancers, including LIHC (Figure 6A). Furthermore, its expression levels in patients with stage I or above (in both Pathologic and Clinical stages) were significantly higher compared to those at stage I (Figure 6B,C). A deeper dive into TCGA's prognostic data and subsequent Kaplan–Meier survival curve plotting revealed that elevated NCAPG2 expression corresponded to poorer Overall Survival and Progress‐Free Interval outcomes, indicating its association with adverse prognosis in HCC patients (Figure 6D,E).

**FIGURE 7:**
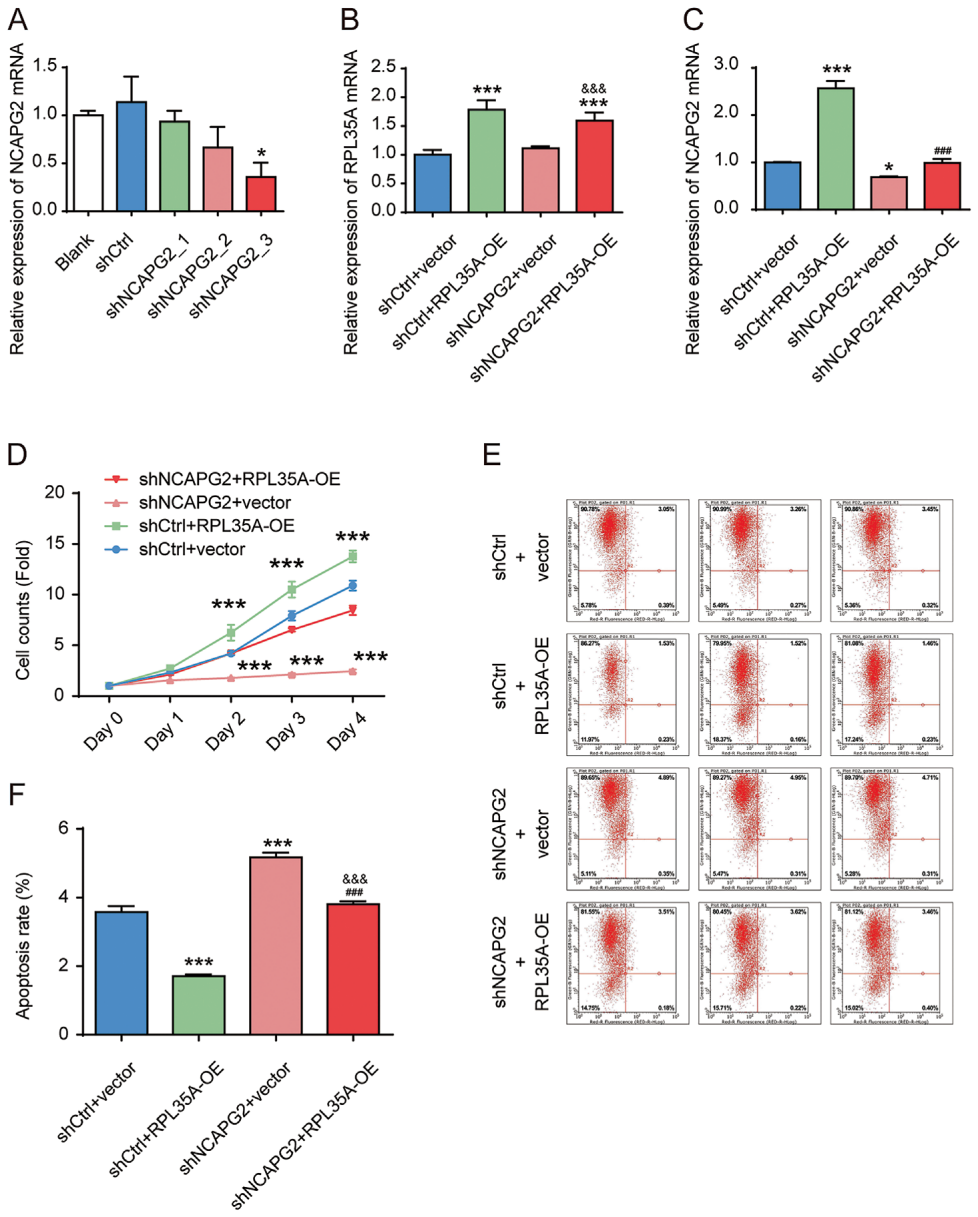
Knockdown of NCAPG2 rescued the proliferation‐promoting effect induced by overexpression of RPL35A in SK‐HEP‐1 cells. (A) BEL‐7404 cells were transfected with three different shRNAs, and the effectiveness of NCAPG2 knockdown was assessed through RT‐qPCR. (B) The expression of RPL35A in BEL‐7404 cells was examined following transfection with NCAPG2 shRNA or the RPL35A overexpression vector. (C) The expression of NCAPG2 in BEL‐7404 cells was measured following transfection with NCAPG2 shRNA or the RPL35A overexpression vector. (D) Cell viability was quantified using the Celigo method, demonstrating the impact of NCAPG2 and RPL35A on BEL‐7404 cells. (E) Flow cytometry was employed to determine the apoptosis rate in BEL‐7404 cells with varying levels of NCAPG2 and RPL35A expression. (F) Statistical analysis of the apoptosis rate in BEL‐7404 cells was provided. *p < 0.05, ***p < 0.001 compared to shCtrl+Vector group. ###*p* < 0.001 compared to shCtrl+RPL35A_OE group. &&&*p* < 0.001 compared to shNCAPG2 + vector group.

**FIGURE 8:**
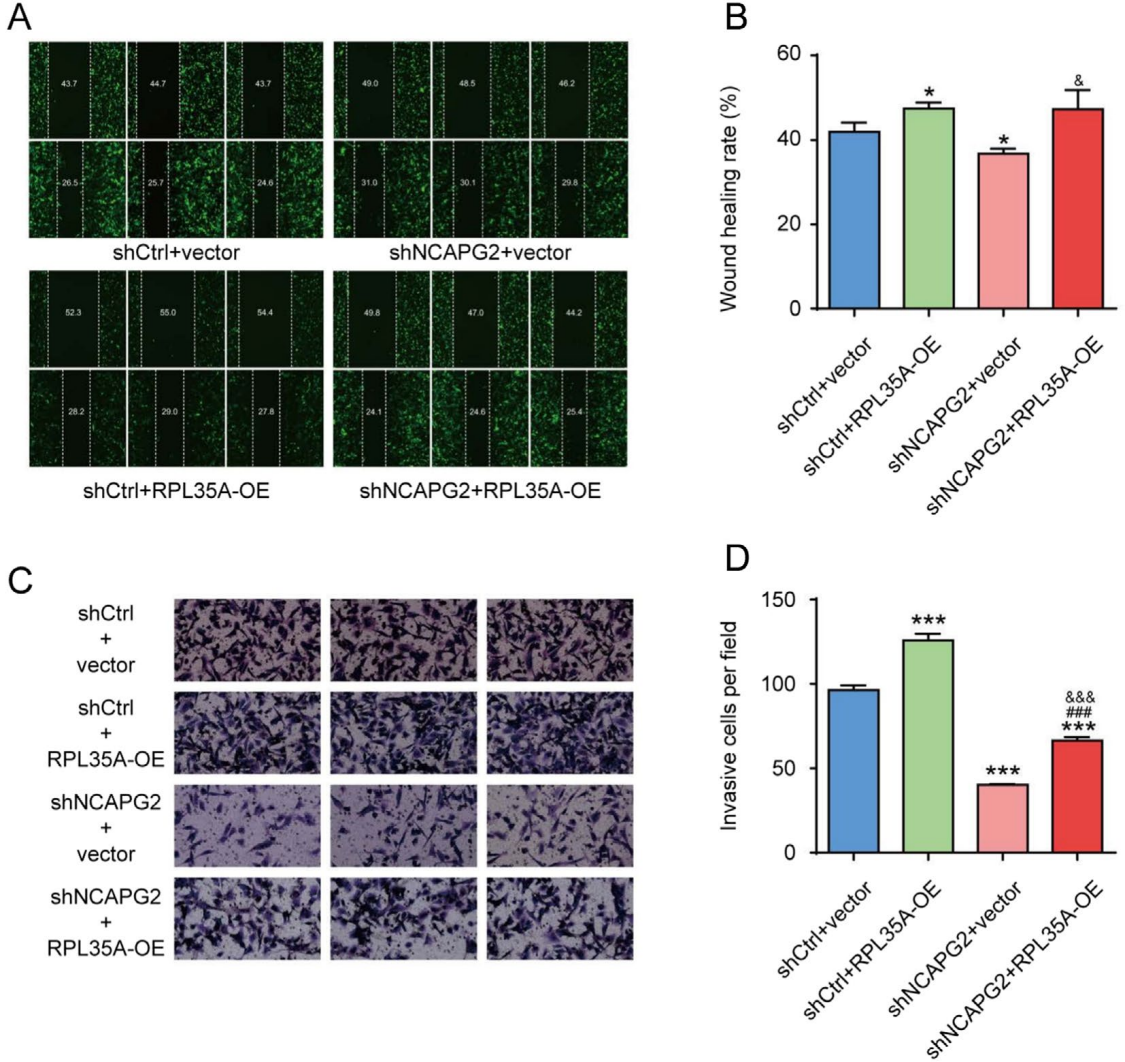
Knockdown of NCAPG2 rescued the metastasis‐promoting effect induced by overexpression of RPL35A in SK‐HEP‐1 cells. (A) Cell migration was assessed using the wound healing method, illustrating the influence of NCAPG2 and RPL35A on SK‐HEP‐1 cells. (B) Wound healing rates were quantified to demonstrate the effect of NCAPG2 and RPL35A on cell migration. (C) Cell invasion was evaluated using the Transwell method, revealing the influence of NCAPG2 and RPL35A on SK‐HEP‐1 cells. (D) The number of infiltrated cells was quantified to illustrate the impact of NCAPG2 and RPL35A on cell migration. *p < 0.05, ***p < 0.001 compared to shCtrl+Vector group. ###*p* < 0.001 compared to shCtrl+RPL35A_OE group. &*p* < 0.05, &&&*p* < 0.001 compared to shNCAPG2 + vector group.

After verifying the knockdown efficiency of NCAPG2 shRNA in SK‐HEP‐1 cells, we selected shRNA_3 for subsequent experiments (Figure 7A). Additionally, we modulated RPL35A expression in cells by constructing and introducing an overexpression lentivirus for RPL35A. A rescue experiment group was also set up, where cells were co‐infected with both the RPL35A overexpression lentivirus and the shNCAPG2 lentivirus. Cells were harvested 72 h post‐transfection, and the expression of RPL35A was determined via RT‐QPCR. We observed a significant increase in RPL35A expression following infection with the overexpression lentivirus, and this upregulation was not affected by the transfection of the shNCAPG2 lentivirus (Figure 7B). Conversely, overexpressing RPL35A markedly elevated NCAPG2 levels in cells, which was reversed upon co‐infection with shNCAPG2 (Figure 7C). The downregulation of NCAPG2 suppressed cell proliferation and enhanced cell apoptosis, effects that were opposite to those observed with RPL35A overexpression (Figure 7D–F). Additionally, the impact of NCAPG2 knockdown on cell proliferation and apoptosis could be reversed by overexpressing RPL35A. In terms of cell migration and invasion, NCAPG2 knockdown inhibited wound healing and reduced the number of cells traversing the Transwell matrix gel (Figure 8). Conversely, overexpression of RPL35A promoted wound closure and increased the number of cells penetrating the Transwell matrix. Furthermore, RPL35A overexpression also reversed the inhibitory effects of NCAPG2 knockdown on cell migration and invasion.

## 4 Discussion

In our study, we initially corroborated the heightened expression of RPL35A across various cancers, including HCC, using data from the TCGA database. This upregulation was also validated in our own HCC samples through immunohistochemistry of HCC tissue microarrays. The elevated RPL35A levels were significantly associated with reduced survival rates, a pattern consistently observed in both the TCGA database and our HCC tissue array. After employing shRNA lentivirus transfection to knock down RPL35A expression, a noteworthy decrease in proliferation, hindered metastasis in vitro and hindered tumorigenesis in vivo was confirmed when compared to the control group. Utilizing microarray technology in conjunction with a comprehensive analysis of the TCGA HCC datasets, we identified NCAPG2 as a downstream target positively regulated by RPL35A, and elevated NCAPG2 levels in HCC tissues were linked to poor prognosis. Notably, the silencing of NCAPG2 effectively reversed the tumor‐promoting effects induced by RPL35A in HCC cells, underscoring the regulatory association between RPL35A and NCAPG2.

The process of protein synthesis is a highly intricate and tightly controlled mechanism that relies on the precise orchestration of ribosomes and a suite of translation factors. This vital procedure of ribosome biogenesis unfolds within the nucleolus, involving a complex interplay among approximately 80 ribosomal proteins, four ribosomal RNAs, supplementary associated proteins and small nucleolar RNAs [22]. Any disruption in the balance of RPs can lead to irregularities in ribosomal biogenesis, which, in turn, can have detrimental effects on fundamental cellular functions such as survival, growth and proliferation [23, 24]. Studies have substantiated that RPs possess functions that extend beyond their roles in the ribosome. These extra‐ribosomal functions encompass participation in DNA repair, replication, cell proliferation, apoptosis and resistance to chemotherapeutic agents [25, 26]. Intriguingly, research using animal models has uncovered a diverse array of phenotypic outcomes resulting from RP mutations, ranging from stunted growth, organ malformations to even embryonic lethality, underscoring the multifaceted roles of RPs that transcend their involvement in ribosomal structure [27, 28, 29]. Within mammalian cells, an insufficiency of RPs leads to a condition known as ribosomal stress, which can trigger a cascade effect, depleting other RPs and causing adverse consequences for cell survival [30, 31, 32]. Moreover, several RPs have been identified as tumor promoters or tumor suppressors in a variety of human tumors, suggesting their potential utility as biomarkers and therapy targets for diverse cancers [8, 33, 34].

RPL35A, a compact ribosomal protein comprising 110 amino acids, represents one of the 47 vital structural constituents of the 60S ribosomal subunit [18]. Its first discovery in mammalian cells took place in murine erythroleukemia cells, where it exhibited high expression levels [35]. Intriguingly, this expression significantly dwindled during cellular differentiation, mirroring the expression patterns seen in RPS19 during terminal erythroid differentiation [36]. Pathogenic mutations in RPL35A have been widely linked to the onset of Diamond‐Blackfan anemia [19, 20]. Recent investigations have unveiled elevated RPL35A levels as an ominous predictor of poor prognosis in diverse cancers, including HCC [13], gastric cancer [21], neuroblastoma [37, 38] and colorectal cancer [39]. In neuroblastoma, the knockdown of RPL35A has been demonstrated to exert negative regulatory control over the ERK pathway, inhibit HIF1α expression and impede aerobic glycolysis [38]. Additionally, RPL35A has been suggested to interact with DEAD‐box3, potentially modulating the E2F pathway and immune responses in colorectal cancer [39]. Nevertheless, no study has hitherto elucidated the precise effects and the underlying molecular mechanisms of RPL35A in HCC. This study conducts an in‐depth analysis using data from both TCGA database and our proprietary HCC cohorts, corroborating the elevated levels of RPL35A and adverse prognoses of HCC. The depletion of RPL35A in HCC cells results in the inhibition of cellular proliferation, migration and invasion while concurrently promoting apoptosis in vitro. Significantly, HCC cells transfected with RPL35A exhibit an impaired capacity for tumorigenesis compared to the negative control. This compelling evidence underscores RPL35A's role as a facilitator of tumor development in HCC.

NCAPG2, a member of the non‐SMC condensin II complex, plays a pivotal role in chromosome condensation and segregation during mitosis [40]. In the context of cancer, it is well documented that NCAPG2 is often highly expressed and significantly contributes to tumor proliferation, metastasis and invasion [41, 42, 43, 44]. Furthermore, the correlations between NCAPG2 and various immune parameters, such as immune cell infiltration, immune checkpoint genes, tumor mutational burden (TMB) and microsatellite instability (MSI), hint at its potential in guiding immunotherapeutic strategies [45, 46, 47]. Notably, in the case of HCC, elevated NCAPG2 expression drives HCC proliferation and metastasis, primarily through the activation of the STAT3 and NF‐κB/miR‐188‐3p signaling pathways [42]. Furthermore, it has been observed that Brachyury, by promoting the transcription of NCAPG2, plays a tumor‐promoting role in HCC [48]. Conversely, miR‐375 was found to inhibit the malignant behavior of HCC cells by directly targeting and inhibiting the expression of NCAPG2 [49]. In our present study, we employed mRNA microarray analysis to identify the downstream genes regulated by RPL35A. By cross‐referencing these co‐upregulated genes of RPL35A in the TCGA database, we identified NCAPG2 as a potential target of RPL35A. Intriguingly, the downregulation of NCAPG2 resulted in the attenuation of HCC proliferation, migration and invasion. Importantly, these effects could be rescued by the overexpression of RPL35A, which provides compelling evidence for the regulatory relationship between RPL35A and NCAPG2.

In summary, this study delved into the upregulation of RPL35A in HCC and robustly validated its significance in predicting patient outcomes. A comprehensive set of both in vitro and in vivo experiments undeniably established RPL35A's role as an enhancer of HCC. Of particular importance, our investigations identified NCAPG2 as a downstream target, influenced positively by RPL35A, thus shedding light on the mechanisms orchestrated by RPL35A to some extent. These findings not only advance our comprehension of HCC progression but also provide a foundation for the exploration of novel biomarkers and potential therapeutic targets in the ongoing battle against HCC.

## Author Contributions

Liang Chen, Yujie Lin, Yibiao Ye, and Xingxi Luo conceived the ideas. Liang Chen, Yujie Lin, Yu Lai, and Yibiao Ye performed all the experiments. Liang Chen, Yujie Lin, Yu Lai, Tao Chen, Xingxi Luo, and Yibiao Ye analyzed the experimental results and initiated the manuscript writing. Xingxi Luo and Yibiao Ye edited the manuscript and supervised the project administration. All the authors approved the manuscript submission.

## Ethics Statement

The authors have nothing to report.

## Consent

The authors have nothing to report.

## Conflicts of Interest

The authors declare no conflicts of interest.

## Supporting information


**Figure S1.** TCGA analysis for screening out RPL35A‐associated genes according to the expression pattern. (A) A heatmap illustrating the co‐expressed genes that are highly expressed in the RPL35A high‐expression group within HCC samples. (B) GO and KEGG enrichment analyses of the genes co‐expressed with RPL35A. (C) GSEA enrichment analysis of genes exhibiting co‐expression patterns with RPL35A. (D) Spearman correlation analysis of the co‐expression patterns of RPL35A and NCAPG2.

## Data Availability

The data and materials used in this study are available from the corresponding author upon reasonable request.
