# Supplementary figures and images for: RPL35A Downregulation Suppresses Hepatocellular Carcinoma Cell Proliferation via NCAPG2 Inactivation
Source: Cancer Med. 2025 Jun 24;14(12):e70985. doi: 10.1002/cam4.70985 (PMC12185999; doi:10.1002/cam4.70985)

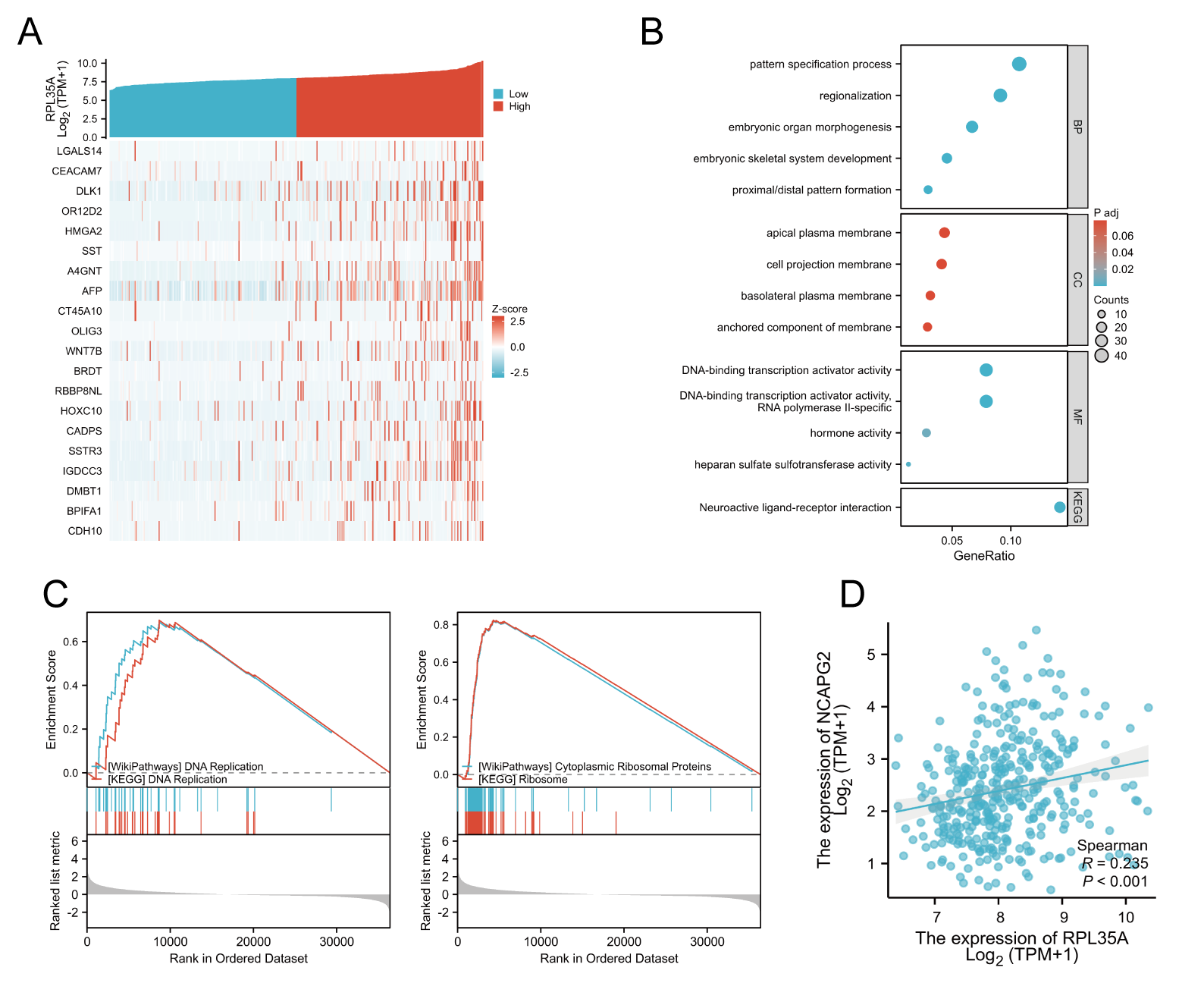

Supplement: Supplementary file 1 — Figure S1. TCGA analysis for screening out RPL35A‐associated genes according to the expression pattern. (A) A heatmap illustrating the co‐expressed genes that are highly expressed in the RPL35A high‐expression group within HCC samples. (B) GO and KEGG enrichment analyses of the genes co‐expressed with RPL35A. (C) GSEA enrichment analysis of genes exhibiting co‐expression patterns with RPL35A. (D) Spearman correlation analysis of the co‐expression patterns of RPL35A and NCAPG2. [file CAM4-14-e70985-s001.tif]
